# Synthesis, characterization and protective efficiency of novel polybenzoxazine precursor as an anticorrosive coating for mild steel

**DOI:** 10.1038/s41598-023-30364-x

**Published:** 2023-04-05

**Authors:** Ahmed M. M. Soliman, Kamal I. Aly, Mohamed Gamal Mohamed, Amer A. Amer, Mostafa R. Belal, Mohamed Abdel-Hakim

**Affiliations:** 1 Department of Chemistry, Faculty of Science, Sohag University, Sohag, 82524 Egypt; 2 Polymer Research Laboratory, Chemistry Department, Faculty of Science, Assiut University, Assiut, 71516 Egypt; 3 Department of Materials and Optoelectronic Science, Center for Functional Polymers and Supramolecular Materials, National Sun Yat-Sen University, Kaohsiung, Taiwan; 4 Chemistry Department, Faculty of Science, Al-Azhar University, Assiut, 71524 Egypt

**Keywords:** Chemistry, Engineering

## Abstract

In this study, 2-[(E)-(hexylimino)methyl] phenol (SA-Hex-SF) was synthesized by adding salicylaldehyde (SA) and n-hexylamine (Hex-NH_2_), which was subsequently reduced by sodium borohydride to produce 2-[(hexylamino)methyl] phenol (SA-Hex-NH). Finally, the SA-Hex-NH reacted with formaldehyde to give a benzoxazine monomer (SA-Hex-BZ). Then, the monomer was thermally polymerized at 210 °C to produce the poly(SA-Hex-BZ). The chemical composition of SA-Hex-BZ was examined using FT-IR, ^1^H, and ^13^C NMR spectroscopy. Differential scanning calorimetry (DSC), thermogravimetric analysis (TGA), scanning electron microscopy (SEM), and X-ray Diffraction (XRD), respectively, were used to examine the thermal behavior, surface morphology, and crystallinity of the SA-Hex-BZ and its PBZ polymer. Mild steel (MS) was coated by poly(SA-Hex-BZ) which was quickly prepared using spray coating and thermal curing techniques (MS). Finally, the electrochemical tests were used to evaluate the poly(SA-Hex-BZ)-coating on MS as anti-corrosion capabilities. According to this study, the poly(SA-Hex-BZ) coating was hydrophobic, and corrosion efficiency reached 91.7%.

## Introduction

**Figure 1:**
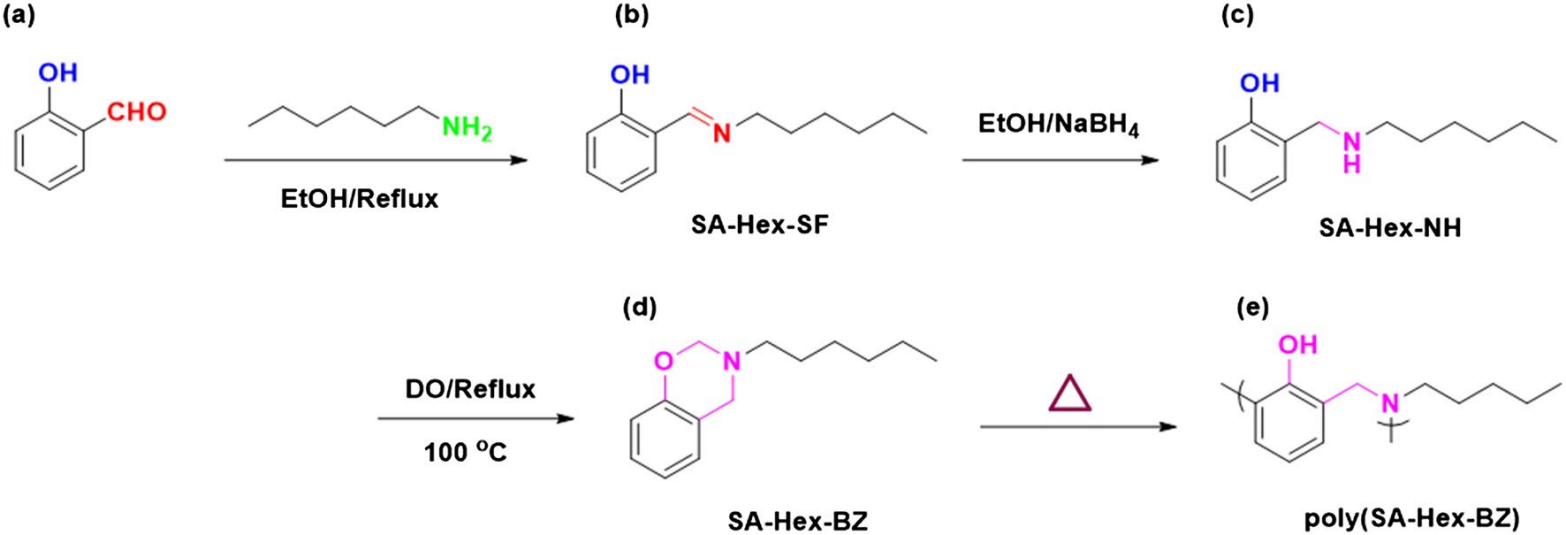
Synthesis of (**b**) SA-Hex-SF, (**c**) SA-Hex-NH, (**d**) SA-Hex-BZ and (**e**) poly(SA-Hex-BZ) from (**a**) SA.

By anticipating the arrival of destructive specialists and working as linked current boundaries, organic coatings were frequently used to resist corrosion in metals and steel^1^. The main strategies for preventing mild steel from unfavorable corrosion during industrial processes include organic coatings that resist corrosion. This keeping in contact with resistance inhibition and the creation of a barrier that prevents the passage of corrosive species and then were thought to be an affordable and practical solution^2,3^. The rates of ion transport and moisture through a coating's polymer network were frequently used to characterize a coating's protective barrier properties^3^. Relatively high-risk PBZ coatings could adhere to metal substrates better and resist corrosion when particular functional groups were incorporated into them^4,5^. Recently, steel surfaces were covered with protective passive oxide layers composed of PBZ-based electroactive species to inhibit corrosion^6,7^. As an analogy, covering mild steel (MS) with curable polybenzoxazine (PBA-ddm) achieved good corrosion inhibition and two-orders of magnitude reduction in the corrosion rate compared to that generated by the uncoated MS^7^. The crosslinking network structure of polybenzoxazines (PBZs) involves intra and intermolecular hydrogen linkages that offered polybenzoxazines many desirable characteristics, outstanding mechanical and insulating characteristics^8,9^, in addition to high thermal stability, high glass transition temperatures, high char yields, almost little shrinkage upon polymerization, low surface free energy, and higher moisture absorption. Benzoxazine monomers were commonly produced via Mannich reactions of phenols, primary amines, and formaldehyde, and could readily polymerize by thermal curing without a catalyst and without releasing any byproducts in their ring-opening polymerization (ROP)^10,11^. High-performance polymers with good mechanical, chemical and thermal properties include PBZs and aromatic polyimides^12^. Various ways were employed to reduce base metal corrosion, among which inhibitors were one of the simplest and utmost well-known^13^. The performance of this monomer and the resultant PBZs might be enhanced by making use of the significant levels of structural flexibility in design and functionalization that were present in benzoxazine monomers. This increased the variety of possible uses for these monomers. When a sulfonic acid unit was inserted into the benzoxazine backbone, for instance, the resulting PBZs exhibited excellent acid resistance and low methanol permeability with good thermal stability in methanol-based fuel cells; they were a good material for hydrogen membranes^14^. The soybean (SE) was utilized to inhibit corrosion on carbon steel in a sulfuric media^15^. PBZ was found to be a promising matrix material, but even in need to be utilized more efficiently in the space environment, it needs to be strengthened against atomic oxygen (AO), ultraviolet (UV), ionizing, vacuum-ultraviolet (VUV), and heat cycles^16,17^. Various materials, particularly polymers, dyes, pigments, and semiconductor devices, were degraded by UV light^18^. Polymeric materials survived permanent deterioration, as a result, impacting their properties^19,20^. Manufacturers used polybenzoxazine coatings, such as electronics, fire resistance, and super hydrophobic coatings at elevated temperatures^21–23^. To increase a polybenzoxazines variety of applications, silane-functionalized polybenzoxazine anticorrosion coating was applied to steel surfaces. This coating effectively decreased the rate of corrosion on steel since the corrosion current was five times lower than that of a pure MS surface^24^. Upon the MS surface, hydrophobic polybenzoxazine (PBA-a) coatings based on bisphenol A were produced. According to studies, the PBA-a coating into MS exhibited superior resistance to corrosion to the epoxy resin coating^7^. P-phenylene diamine benzoxazine and commercial bisphenol A based on benzoxazine also was utilized as a corrosion-resistant coating on 1050 aluminum alloy^25^. Recent studies have shown the efficiency of PBZ derivatives developed from bio-based materials, including vegetable oil, in inhibiting the corrosion of steel covered with Zn–Mg–Al alloy^15,26,27^. These studies revealed that PBZs could be used as corrosive environmental materials^28^. A new type of PBZ precursor called main-chain-type benzoxazine polymer (MCBP) contained cross-linkable benzoxazine rings within the polymer backbone^29^. Using diamine, bisphenol A, as well as paraformaldehyde, high molecular weight PBZ was synthesized^30^. According to the results of toughness tests, higher molecular weight PBZ thermosets produced from MCBP are more durable than any of those prepared from more common, lower molecular weight PBZ. An isomer combination of paraformaldehyde, diamines, and bisphenol-F was used to produce good physical and mechanical characteristics with MCBPs^31^. Pyrimidine derivatives also were reported as an effective ecofriendly corrosion inhibitor in acidic environments^32^. Improvement of mild steel's ability to resist corrosion in an acid environment by using unique carbon dots as a green corrosion inhibitor^33^. Herein, we synthesized novel benzoxazine monomer (SA-Hex-BZ) through Schiff base condensation of n-hexyl amine with SA followed by reduction of Schiff base compound by sodium borohydride and finally, ring closing by formaldehyde in 1,4-dioxane (DO) at 100 °C [Fig. 1], which their chemical structures were proved by FTIR ^1^H and ^13^CNMR. The thermal stabilities, thermal curing behavior, and surface morphology of the SA-Hex-BZ and poly(SA-Hex-BZ) were confirmed by TGA, DSC, and Scanning Electron Microscopy (SEM). On the MS surface, the SA-Hex-BZ monomer was sprayed on and thermally cured. Open-circuit potentials (OCPs) results showed that our poly(SA-Hex-BZ) coating was excellent anti-corrosion performance.

## Experimental section

### Materials

Salicylaldehyde (SA), n-hexylamine (Hex-NH_2_), ethanol, sodium hydroxide (NaOH), anhydrous sodium sulfate, formaldehyde, 1,4-dioxane (DO), chloroform, sodium borohydride (NaBH_4_) and dilute hydrochloric acid were purchased from Acros. All melting points were recorded and corrected on the Melt-Temp II melting point instrument. The chemicals and solvents used in this experiment were all purchased from Sigma-Aldrich and are all of the analytical grades.

### Synthesis of 2-[(E)-(hexylimino)methyl] phenol [SA-Hex-SF]

**Figure 2:**
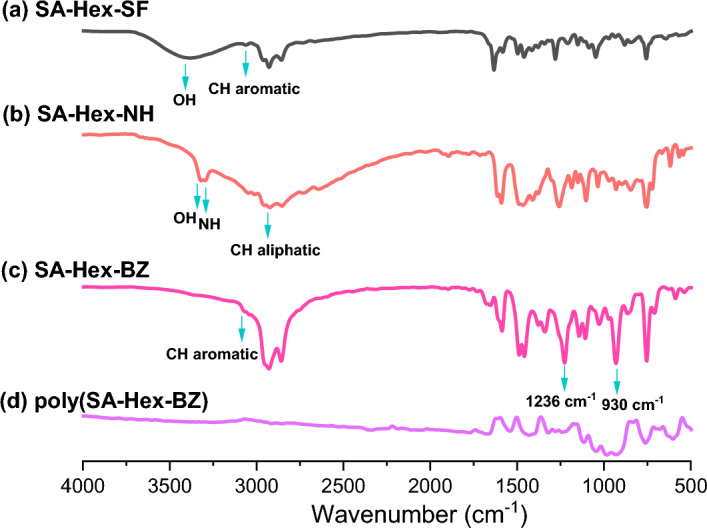
FTIR analyses of (**a**) SA-Hex-SF, (**b**) SA-Hex-NH, (**c**) SA-Hex-BZ and (**d**) poly(SA-Hex-BZ).

**Figure 3:**
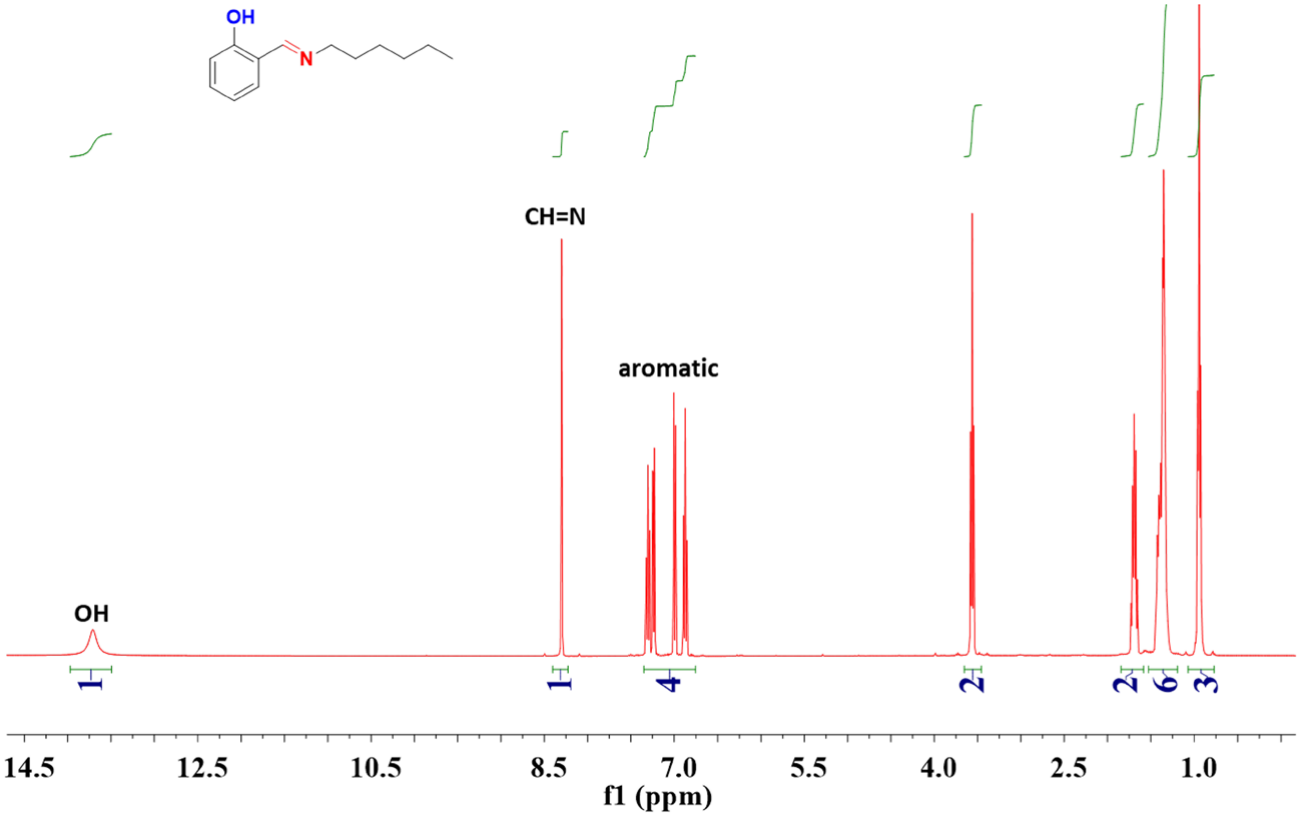
^1^H NMR spectrum of SA-Hex-SF.

**Figure 4:**
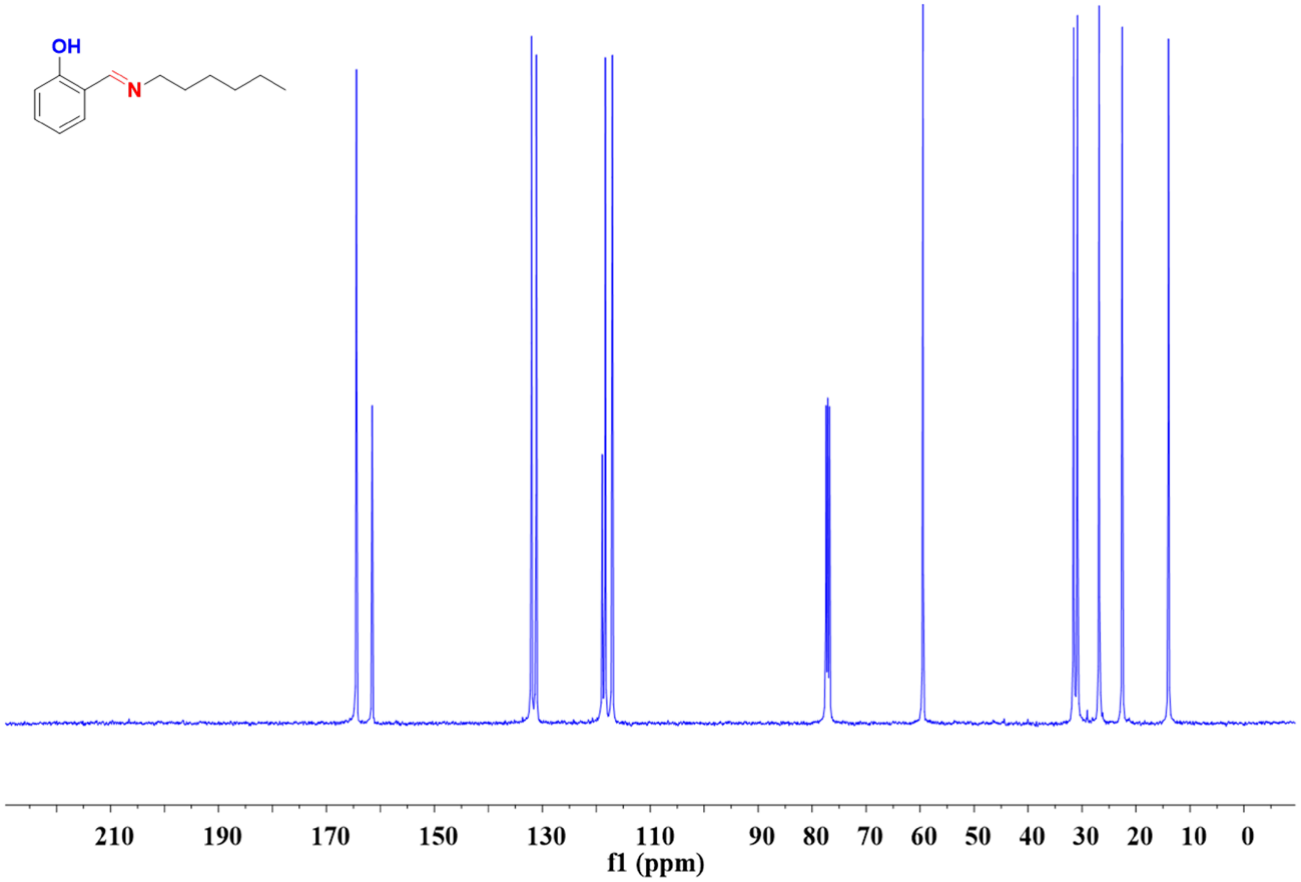
^13^C NMR spectrum of SA-Hex-SF.

Hex-NH_2_ (40 mmol, 5.25 mL) was stirred slowly to a solution of SA (40 mmol, 4.2 mL) in abs. ethanol (30 mL) for 5 h at 60 °C. The viscous liquid product was transparent and yellow in color [Fig. 1b]. b.p: 78–79 °C. FTIR (KBr, cm^−1^, Fig. 2a): 3550–3300 (OH), 1633 (CH=N).^1^H-NMR (400 MHz, CDCl_3_, δ, ppm, Fig. 3): 13.85 (s, 1H, -OH), 8.35 (s, 1H, –CH=N–), 6.80–7.40 (m, 4H, ArH), 3.60 (t, 2H, –CH_2_–), 1.70 (m, 2H, –CH_2_–), 1.40 (m, 4H, 3–CH_2_–), 1.0 (t, 3H, –CH_3_), ^13^C-NMR (100 MHz, CDCl_3_, δ, ppm, Fig. 4): on decoupled 163 (–CH=N–), 120–161 (6C aromatic) which become (4C aromatic) on dept due to 2C have no proton.

### Synthesis of 2[(hexylamino)methyl]phenol [SA-Hex-NH]

**Figure 5:**
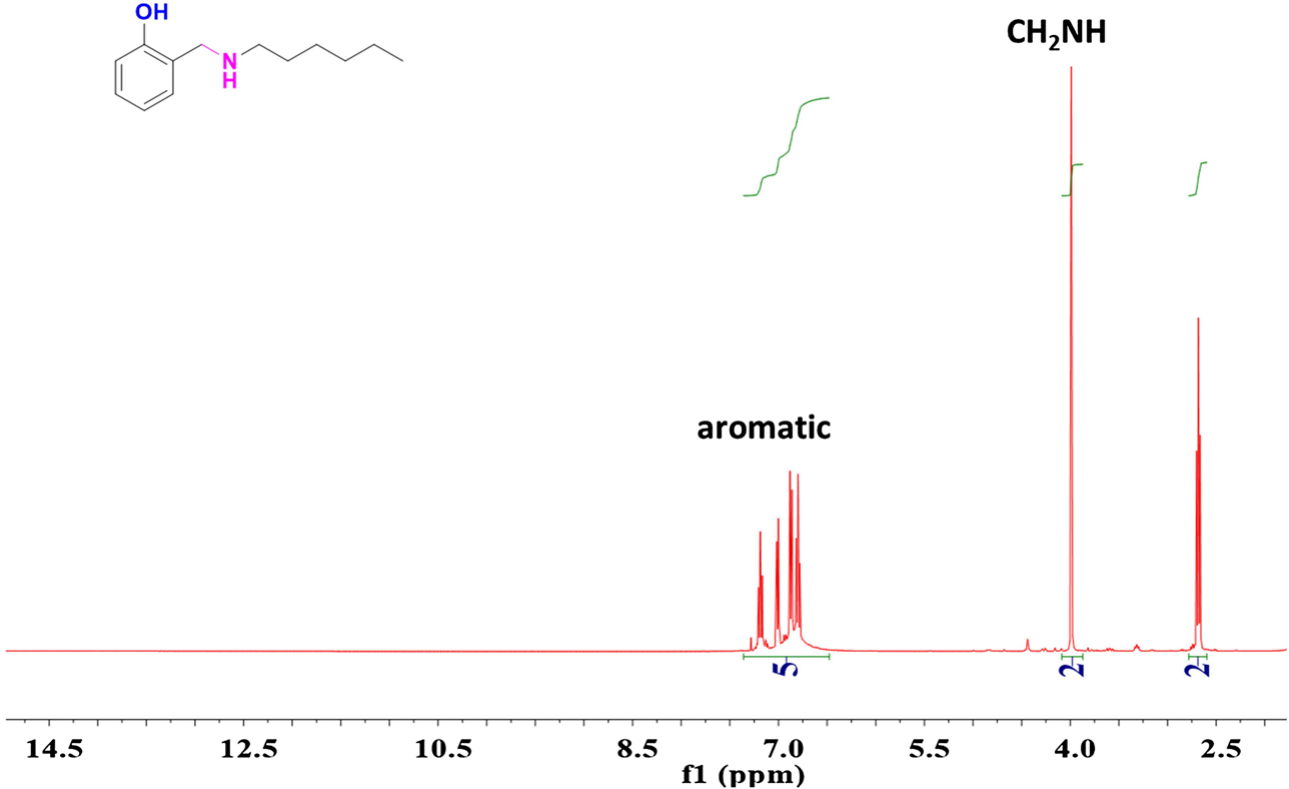
^1^H NMR spectrum of SA-Hex-NH.

SA-Hex-NH (20 mmol, 4.10 g) and excess NaBH_4_ (0.76 g) were added slowly with stirring for 3 h at room temperature. Then, 100 mL of water was added when the reduction was completed, and the product was extracted with chloroform, washed with water, dried over anhydrous sodium sulfate, and concentrated until dry. The viscous liquid product tas transparent and yellow in color [Fig. 1b]. FTIR (KBr, cm^−1^, Fig. 2b): 3212 (NH, stretching), 3100–3300 (OH, broad) due to inter and intramolecular hydrogen bond, 1572 (–NH–, bending). ^1^H-NMR (400 MHz, CDCl_3_, δ, ppm, Fig. 5): 7.80 (s, 1H, OH), 6.77–7.30 (m, 4H, ArH), 4.0 (s, 2H, –CH_2_–), 2.70 (t, 2H, –CH_2_–), 1.55 (m, 2H, –CH_2_–), 1.35 (m, 6H, 3–CH_2_–), 0.5 (t, 3H, –CH_3_).

### Synthesis of n-hexylamine based-benzoxazine (SA-Hex-BZ)

**Figure 6:**
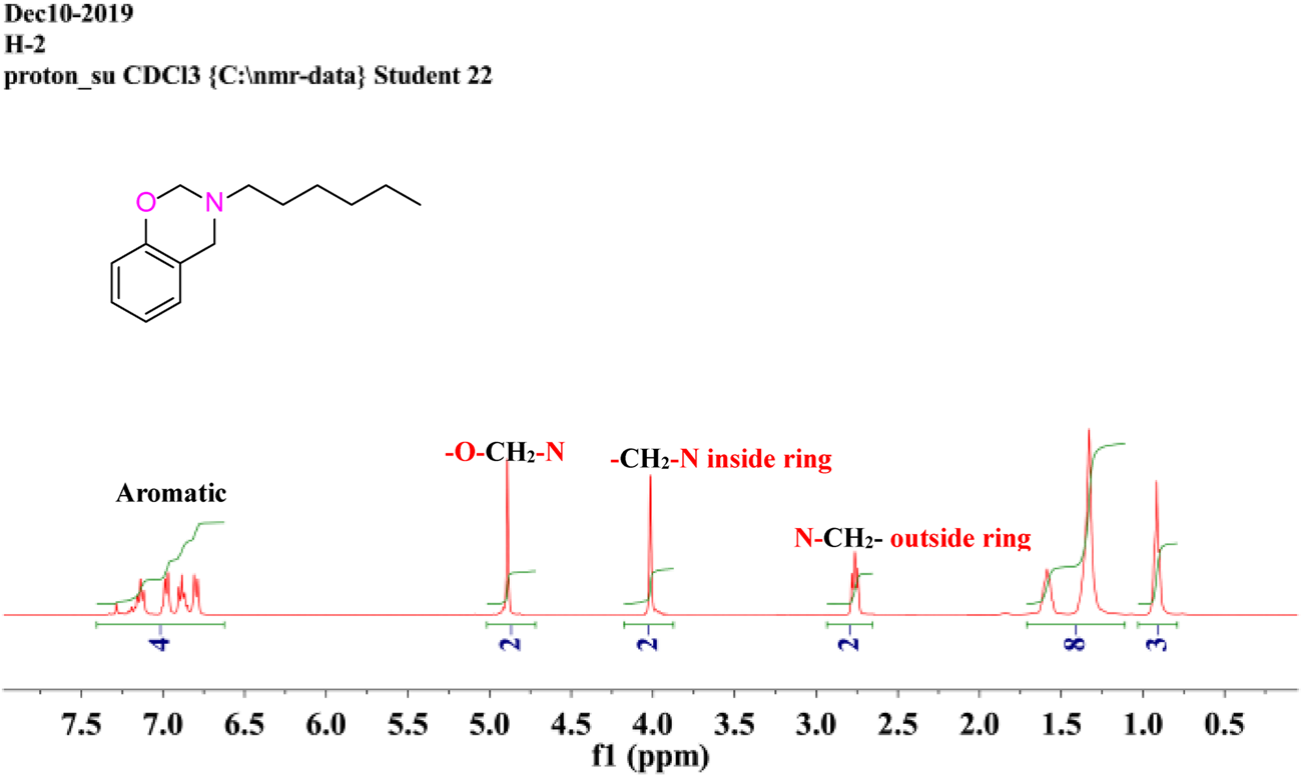
^1^H NMR spectrum of SA-Hex-BZ.

**Figure 7:**
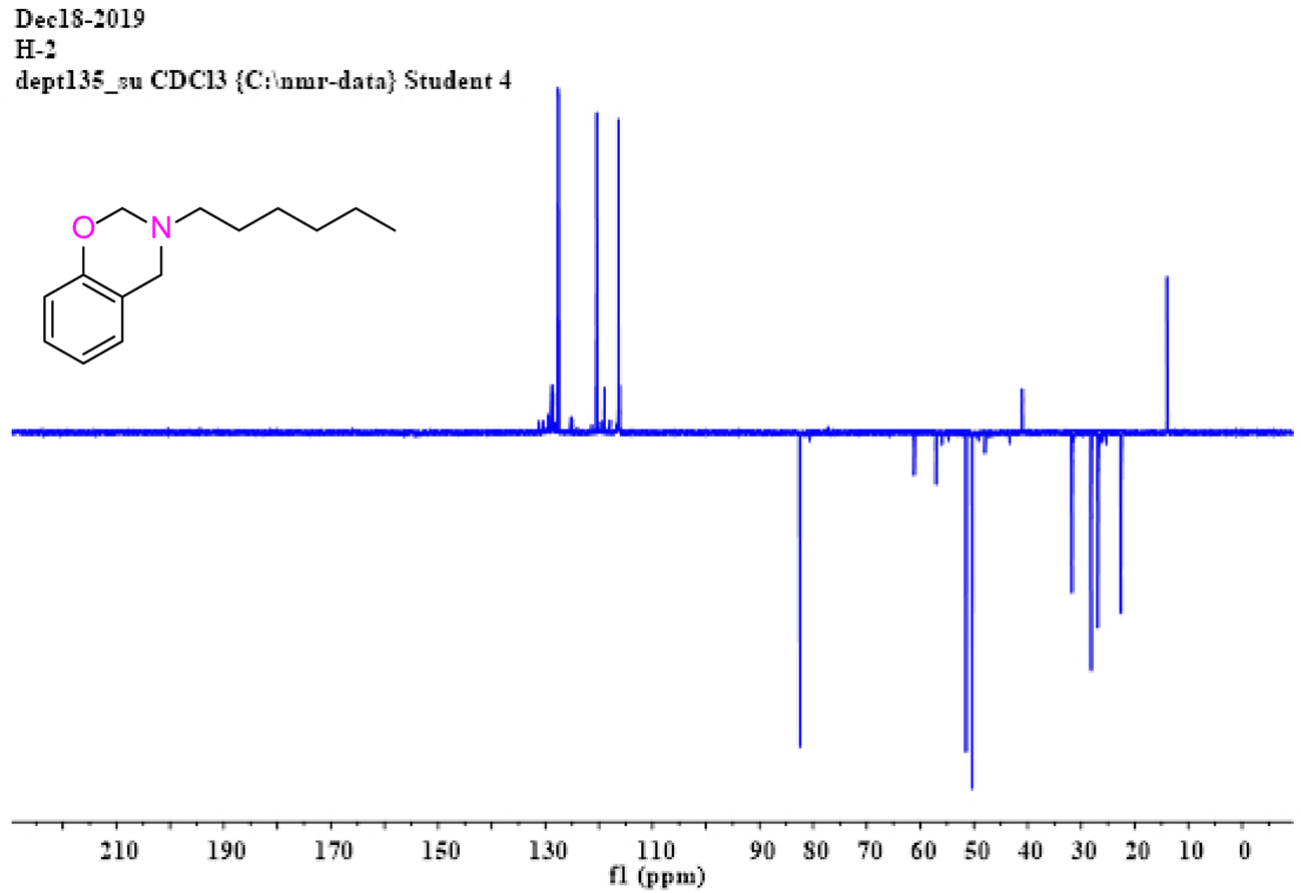
^13^C NMR spectrum of SA-Hex-BZ.

SA-Hex-NH (30 mmol, 6.21 g) was stirred with an excess of formaldehyde (32 mmol, 1.14 mL) in 30 mL of DO at 100 °C for 27 h. The residue was dissolved in chloroform and washed with NaOH (20 mL, 2 M) solution just after the solvent had already been evaporated. Over sodium sulfate, the organic layer was dried and extracted to dryness. The product was a brownish liquid oil [Fig. 1d]. The physical properties of SA-Hex-Bz are listed in Table 1. FTIR (KBr, cm^−1^, Fig. 2c), 1236 (COC antisymmetric stretching), 1107 (COC symmetric stretching), and 930 (oxazine ring). ^1^H-NMR (400 MHz, CDCl_3_, δ, ppm, Fig. 6): 6.75–7.40 (m, 4H, ArH), 4.90 (s, 2H, OCH_2_N), 4.0 (s, 2H, ArCH_2_N=), 2.20 (t, 2H, –CH_2_–), 1.50 (m, 8H, 4–CH_2_–), 0.95 (t, 3H, –CH_3_). ^13^C NMR (100 MHz, CDCl_3_, δ, ppm, Fig. 7): 129.93–118.12 (aromatic), 84.63 (-OCH_2_N-), 51.41 (ArCH_2_N-).

**Table 1:** Physical properties for SA-Hex-BZ and poly(SA-Hex-BZ).

Physical properties	SA-Hex-BZ	Poly(SA-Hex-BZ)
State	Oily liquid (resin), boiling point = 179 °C	Solid
Color	Brown	Black
Miscibility	Immiscible	Insoluble
Solvent	Chloroform	Insolube in toluene, xylene, benzene, chloroform, ethanol, methanol, formic or acetic

### Preparation of poly(SA-Hex-BZ)

In accordance with the procedure for the synthesis of poly(SA-Hex-BZ), the SA-Hex-BZ monomer was cured in a furnace at 210 °C; for 2 to afford poly(SA-Hex-BZ) as a black solid, as presented in Fig. 1e. The physical properties of poly(SA-Hex-BZ) are listed in Table 1.

### Preparing studied surface and tested media

0.17% C, 0.072% Ni, 0.022% Si, 0.0017% Al, 0.011% Mo, 0.010% P, 0.71% Mn, 0.182% Cu, 0.022% F, 0.045% Cr, 0.011% Sn and 98.74% Fe constitute the mild steel (MS) specimen^34^. We cut the MS specimens into 1 × 1 × 1 cm^3^ blocks for the electrochemical measurements. Every specimen placed through the testing process has its surfaces cleaned with acetone first, then polished with emery polishing paper of different grades, including 1200 and 1400, before even being dried. The corrosive solutions are made with analytical grade 97% H_2_SO_4_ (Sigma-Aldrich Laborchemikalien, Germany) and are subsequently diluted with bi-distilled water before usage.

### Corrosion tests

The SA-Hex-BZ and poly(SA-Hex-BZ) were dissolved in 200 ppm chloroform to create the inhibitor's solution. The method employed in the studies involves spraying monomer on the surface of MS and curing it at 210 °C for two hours. The resultant layer has a 4-µm thickness that forms a thin coating (poly(SA-Hex-BZ)) on the mild steel electrode (using a micrometer caliper). The corrosion-causing medium must be immersed to allow the open circuit potential to proceed.

### Characterization

FTIR spectra were recorded using a Bruker Tensor 27 FTIR spectrophotometer with a resolution of 4 cm^–1^ through the KBr disk method. ^13^C Nuclear magnetic resonance (NMR) spectra were recorded using an INOVA 500 instrument with CDCl_3_ as the solvent and TMS as the external standard; chemical shifts were reported in parts per million (ppm). The thermal stabilities of the samples were examined under an N_2_ using a TG Q-50 thermogravimetric analyzer; each cured sample (ca. 5 mg) was placed in a Pt cell and heated at a rate of 20 °C min^–1^ from 100 to 800 °C under a N_2_ flow rate of 60 mL min^–1^. Wide-angle X-ray diffraction (WAXD) patterns were measured using the wiggler beamline BL17A1 of the National Synchrotron Radiation Research Center (NSRRC), Taiwan; a triangular bent Si (111) single crystal was used to give a monochromated beam having a wavelength (*λ*) of 1.33 Å. The morphologies of the samples were examined using field emission scanning electron microscopy (FE-SEM; JEOL JSM7610F). X-ray photoelectron spectroscopy (XPS) was collected on K-ALPHA (Thermo Fisher Scientific, USA) with monochromatic X-ray Al K-alpha radiation − 10 to 1350 eV spot size 400 micron at pressure 9–10 bar with full spectrum pass energy 200 eV and at narrow spectrum 50 eV. The Raman spectra were investigated using Horiba Jobin–Yvon HR800 Raman Spectrometer with 633 nm laser, 10 s accumulated scans repeated 20 times, and a 50× magnification lens.

## Results and discussion

### Thermal curing behavior of SA-Hex-BZ monomer to produce poly(SA-Hex-BZ)

**Figure 8:**
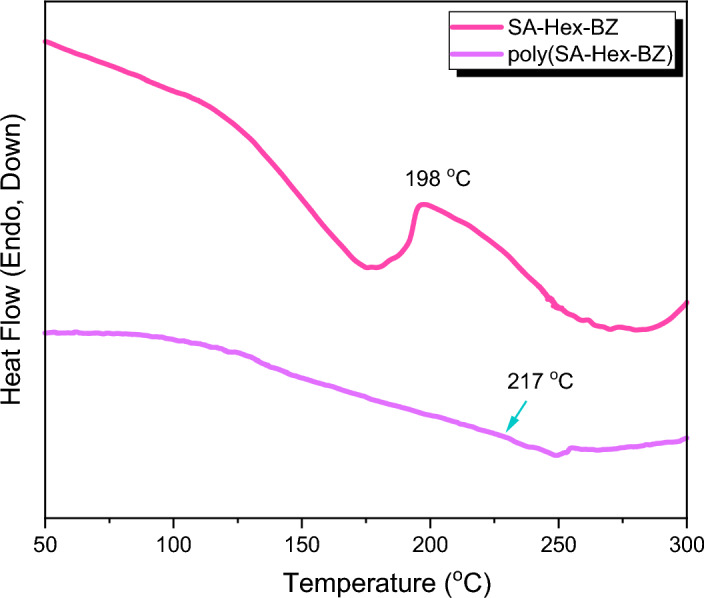
DSC thermograms of SA-Hex-BZ and poly(SA-Hex-BZ).

**Figure 9:**
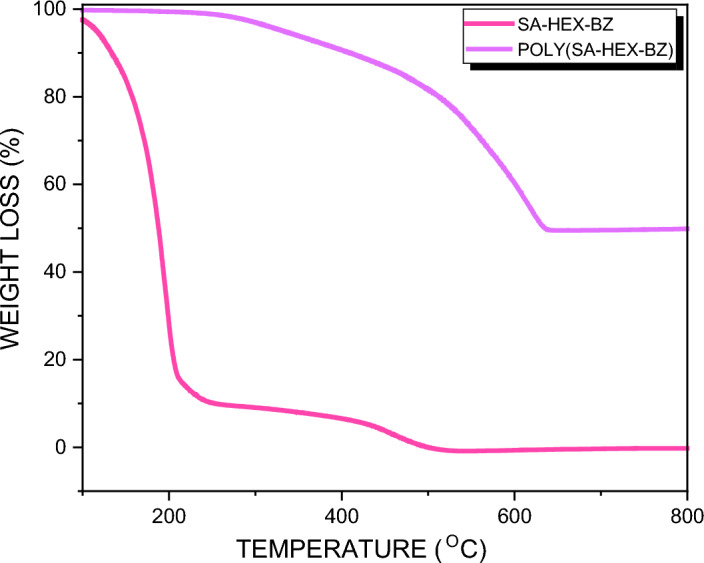
TGA profiles of SA-Hex-BZ and poly(SA-Hex-BZ).

**Figure 10:**
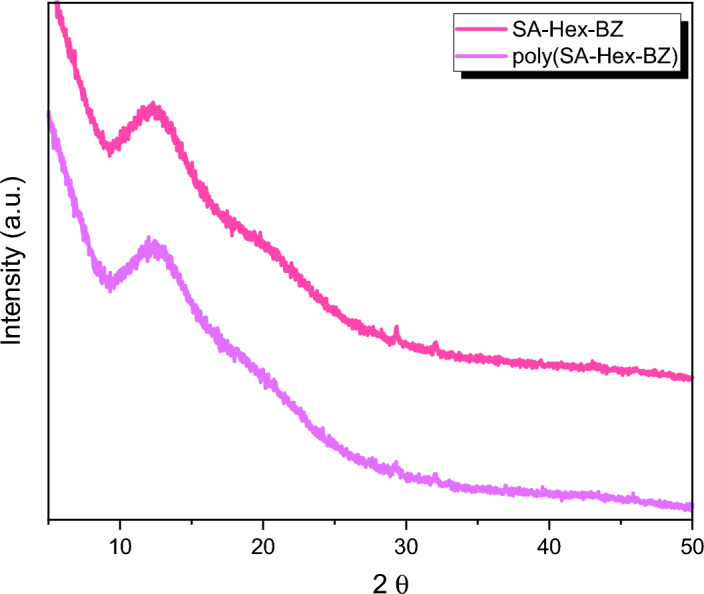
XRD Patterns of SA-Hex-BZ and poly(SA-Hex-BZ).

**Figure 11:**
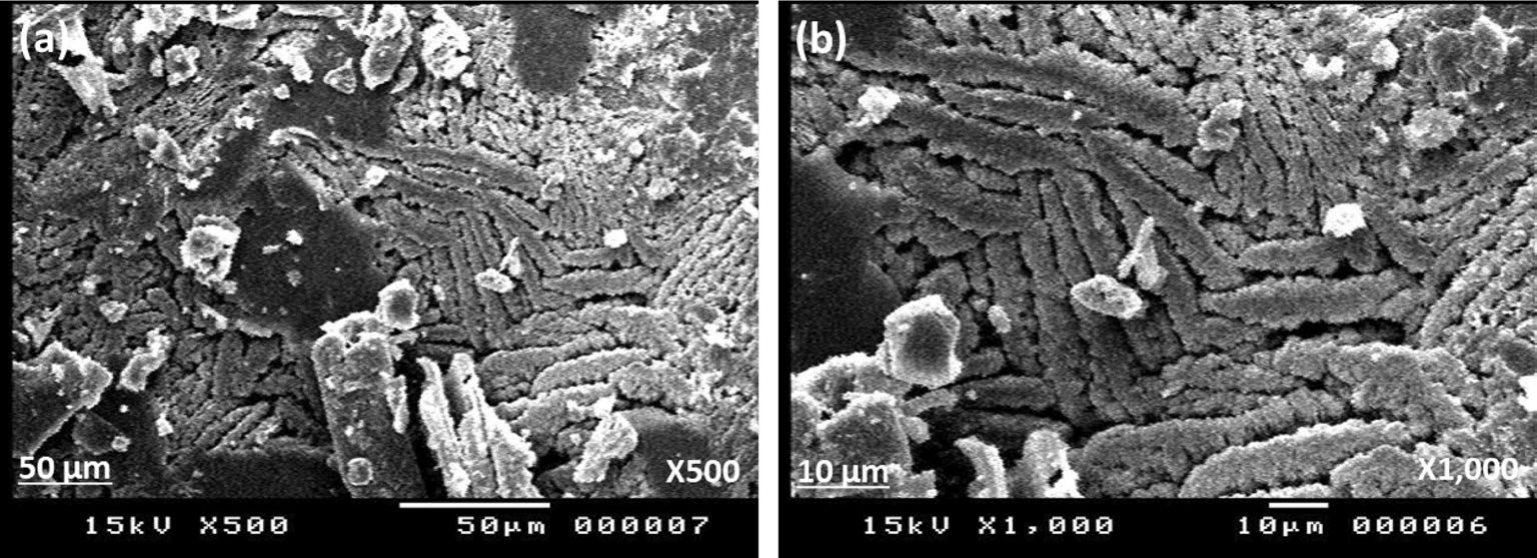
SEM images of poly(SA-Hex-BZ) ad different magnification (×500 (**a**) and ×1000 (**b**)).

Herein, salicylaldehyde (SA) [Fig. 1a] and n-hexylamine (Hex-NH_2_) were used to produce SA-Hex-SF [Fig. 1b], which was then reduced by NaBH_4_ to afford SA-Hex-NH [Fig. 1c], and finally the SA-Hex-NH reacted with CH_2_O to afford a new benzoxazine precursor called SA-Hex-BZ) [Fig. 1d]. Then, the poly(SA-Hex-BZ) was prepared through thermal curing polymerization of its monomer at benzoxazine at 210 °C [Fig. 1e]. The FTIR spectrum of SA-Hex-BZ features characteristics absorption signals centered at 1236 and 1107 cm^−1^, corresponding to asymmetric and symmetric C–O–C stretching, respectively, which occurs slight disappearance or changes in its intensity, as well as the intensity of peak for OH group [Fig. 2d], was increased, subsequent thermal curing 210 °C to give poly(SA-Hex-BZ). The FT-IR analysis confirmed the ring-opening polymerization of the SA-Hex-BZ monomer, which revealed that the oxazine ring's absorption band at 933 cm^−1^ practically vanished after thermal curing. Figure S1 presents the Raman profile of poly(SA-Hex-BZ) after thermal polymerization of SA-Hex-BZ monomer at 210 °C. As observed, The bands (1236, 1107, and 930 cm^−1^) from the benzoxazine ring are consumed during the ring opening reaction of the SA-Hex-BZ monomer. The peak at 1611 cm^−1^ attributed to the C=C from the benzene ring still existed and was used as an internal standard because the aromatic ring is not consumed during the thermal polymerization of the BZ monomer. The peaks of C1*s*, N1*s*, and O1*s* in the poly(SA-Hex-BZ) sample were found at 285.32 eV, 400.07 eV, and 532.97 eV; respectively, based on XPS analysis [Figure S2]. Figure 8 shows DSC thermograms of the SA-Hex-BZ and poly(SA-Hex-BZ). As shown in the DSC profile of SA-Hex-BZ, the endothermic peak may be attributed to the melting point, and the exothermic peak (ROP) was 179 °C and 198 °C, respectively^35^. As compared to prior imide-functionalized benzoxazine investigations that had been employed in the manufacture of high-performance materials, this study's exothermic peaks manifested at lower temperatures^22^. After curing of SA-Hex-BZ at 210 °C, the exothermic peaks of SA-Hex-BZ entirely vanished, suggesting full ROP. Additionally, the glass transition temperature (T_*g*_) of poly(SA-Hex-BZ) after curing at 210 °C was computed in Table 2, at which the T_g_ value of poly(SA-Hex-BZ) was 217 °C. These results are the average from three different observations. Hence, the T_*g*_ value in our new poly(SA-Hex-BZ) was higher compared to the cross-linked materials reported NDOPodaBz (205 °C after curing at 210 °C)^36^. We were able to explain the high value of T_*g*_ of poly(SA-Hex-BZ) by referring to the high density of inter- and intra-molecular hydrogen bonds between the phenolic OH groups and the nitrogen atoms in Mannich bridges. The stability of the SA-Hex-BZ monomer as well as its corresponding poly(SA-Hex-BZ) obtained after curing at 210 °C was studied using TGA (Fig. 9, Table 2). We considered the temperature for 5%, 10%, and 50% weight loss as (T_d5_ and T_*d10*_, T_*d50*_, respectively). As the curing temperature increased, highly cross-linked thermosets developed, increasing the data of T_*d5*_, T_*d10*_, T_*d50*_, and char yields. After curing the monomer at 210 °C, the data of T_*d5*_, T_d10_, T_d50,_ and char yields at 800 °C have been 332, 409, 635 °C, and 51.89 wt.%, respectively; for SA-Hex-BZ monomer data of T_*d5*_, T_*d10*_, T_*d50*,_ and the char yields have been 116, 134, 188 °C, and 0.85 wt.%, respectively. Thus, the thermal stabilities of our new poly(SA-Hex-BZ) were higher than that of the SA-Hex-BZ. The XRD profiles of the SA-Hex-BZ and poly(SA-Hex-BZ) obtained after thermal curing at 210 °C showed that the signal at 2 = 11° refers to the (002) plane, which represents irregular and amorphous carbons [Fig. 10]. The SEM images [Fig. 11] of poly(SA-Hex-BZ), after thermal curing at 180 and 210 °C, the SEM images displayed that the poly(SA-Hex-BZ) particles were placed next to each other like ropes.

**Table 2:** DSC and TGA results for SA-Hex-BZ and poly(SA-Hex-BZ).

Samples	Curing temperature (°C)	*T*_d5_ (°C)	*T*_d10_ (°C)	*T*_d50_ (°C)	Char yield (%)	*T*_g_/DSC (°C)
SA-Hex-BZ	25	116	134	188	0.85	–
poly(SA-Hex-BZ)	210	332	409	635	51.89	217

## Electrochemical techniques

### Open circuit potential

**Figure 12:**
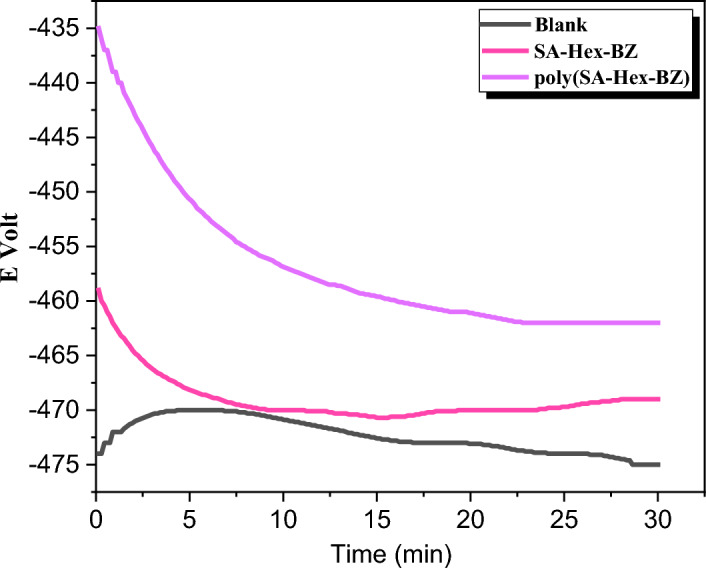
Eocp–time plots of uncoated MS and MS coated with SA-Hex-BZ and poly(SA-Hex-BZ).

Figure 12 shows the curves of E (mV) vs. time (min.) at current zero for MS immersed in the blank solution and 200 ppm of the tested inhibitors (SA-Hex-BZ and poly(SA-Hex-BZ). It was clear that for blank solution curves, E_s.s_ moves to a negative potential than E_im_. This change is owing to the oxide film deteriorating from the surface of MS until it enters the corrosion cell's Es.s. The addition of various concentrations of the testing inhibitors resulted in a shift of E_s.s_ value to a more positive potential than the uncoated MS. The latter result from such an inhibitor molecule layer being adsorbed on the MS surface's active sites. Data derived from open circuit potential (OCP) are displayed in Table 3.

**Table 3:** Potential (mV) of mild steel exposed to 1.0 M H_2_SO_4_ with SA-Hex-BZ coating and poly(SA-Hex-BZ) coating against time (min).

Inhibitors	− E_im_	− E_s.s_
1.0 M H_2_SO_4_	474 ± 1.4	475 ± 1.2
SA-Hex-BZ	459 ± 1.3	469 ± 1.4
Poly(SA-Hex-BZ)	435 ± 0.9	462 ± 1.1

### Tafel polarization

The Tafel plot polarisation method estimates the corrosion current, potential, rate, and inhibition efficiency of mild steel that has been subjected to acidic conditions both with and without inhibitors within a range of 250 mV versus ES.S and at a scan rate of between 0.166 and 0.3 mV/S. The corrosion rate (denoted by CR) and the inhibitor efficiency percent (IE %), can be determined using Eqs. (1) and (2).^37^

1 $$\mathrm{CR}= \frac{0.13\times \mathrm{Icorr}\times \mathrm{Eq}.\mathrm{Wt}}{\uprho \times \mathrm{A}}$$

where CR was the (corrosion rate mpy), I_corr_ was the (corrosion current density μA/cm^2^), which records the current value at which the corrosion process takes place, Eq. Wt. was the equivalent weight of the metal (g/equivalent) equal to 55.8 atomic mass, A is the area (cm^2^) immersed in tested solutions, ρ is the density (gm/cm^3^) equal to 7.874 g/cm^3^, and 0.13 was the metric and time conversion factor.

2 $$\mathrm{IE}\%=\frac{\mathrm{CR}-\mathrm{CR}1}{\mathrm{CR}} \times 100$$

**Figure 13:**
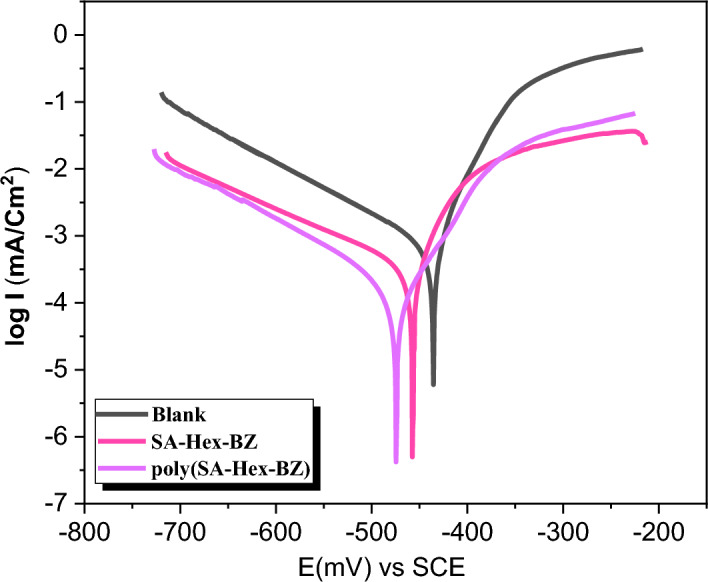
Tafel plots of uncoated MS and MS coated with SA-Hex-BZ and poly(SA-Hex-BZ).

Figure 13 shows potentiodynamic polarisation curves of mild steel corrosion in 1.0 M H_2_SO_4_ solution with and without inhibitors. It was observed that the presence of SA-Hex-BZ and poly(SA-Hex-BZ), caused shifting in Tafel slopes. This indicated that: (1) the adhesion of inhibitor molecules to the MS electrodes' surface and (2) the E_corr_ of used inhibitors differs positively from that of the blank solution, and the difference doesn't reach 85 mV, which proved that these inhibitors are mixed ones and are reduced in anodic and cathodic Tafel slope^38^. Table 4 records the parameters extracted from TF such as I_corr_, E_corr_, CR, IE%, and θ of MS with and without inhibitors. In the absence of studied inhibitors, I_corr_ increased to reach 2990 (µA/cm^2^), and CR increased to reach 2488 mpy. In addition, with the inhibitors to the blank solution, a decrease in each of I_corr_, CR, and IE% was observed. The poly(SA-Hex-BZ) exhibits more inhibition efficiency than the SA-Hex-BZ monomer (91.7% and 84.4%, respectively). The high inhibition efficiency of poly(SA-Hex-BZ) (91.7%) compared with SA-Hex-BZ monomer (84.4%) was caused, upon ring-opening of the oxazine units, there was a higher cross-linking density and intra–intermolecular hydrogen bonding in the earlier. The corrosion behavior of MS in 1.0 M H_2_SO_4_ solution in the absence and presence of poly(SA-Hex-BZ) was investigated by the electrochemical impedance spectroscopy (EIS) method at 25 °C. Nyquist plots of MS in 1.0 M H_2_SO_4_ solution with and without the poly(SA-Hex-BZ) is given in Figure S3, which shows a single semicircle and reveals that the charge transfer process is occurring at the electrode/solution interface. The addition of the poly(SA-Hex-BZ) does not change the impedance shape, but the diameter of this semicircle increases in the presence of poly(SA-Hex-BZ) than in its absence. It is clear that the impedance response for steel in HCl changes significantly in the presence of poly(SA-Hex-BZ) and the corrosion of steel is inhibited in the presence of the poly(SA-Hex-BZ). Figure S4 displayed the surface morphology of poly(SA-Hex-BZ) coating on mild steel after curing SA-Hex-BZ monomer at 210 °C for 2 h and immersing coated MS with poly(SA-Hex-BZ) in corrosive solution 1.0 M H_2_SO_4_ solution. The SEM images revealed that there are no defects like pores or cracks appearing in the morphology of poly(SA-Hex-BZ) coatings after the corrosion process. Moreover, The surface is lightly damaged, which indicates that poly(SA-Hex-BZ) coating on MS can effectively inhibit the corrosion of MS in the aggressive H_2_SO_4_ solution. This emerged from coordination interaction among poly(SA-Hex-BZ) coatings and MS surfaces, that have high adsorbing properties.

**Table 4:** Parameters of mild steel's potentiodynamic polarisation in 1.0 M H_2_SO_4_ with SA-Hex-BZ coating and Poly (SA-Hex-BZ) coating.

Inhibitors	I (µA/Cm^2^)	C.R	IE%	θ
1.0 M H_2_SO_4_	2699 ± 0.10	2489 ± 0.1	–	–
SA-Hex-BZ	456 ± 0.15	420 ± 0.15	84.4 ± 0.15	0.84 ± 0.15
poly(SA-Hex-BZ)	243 ± 0.01	224 ± 0.01	91.7 ± 0. 01	0.92 ± 0.01

### Corrosion protection mechanism

**Figure 14:**
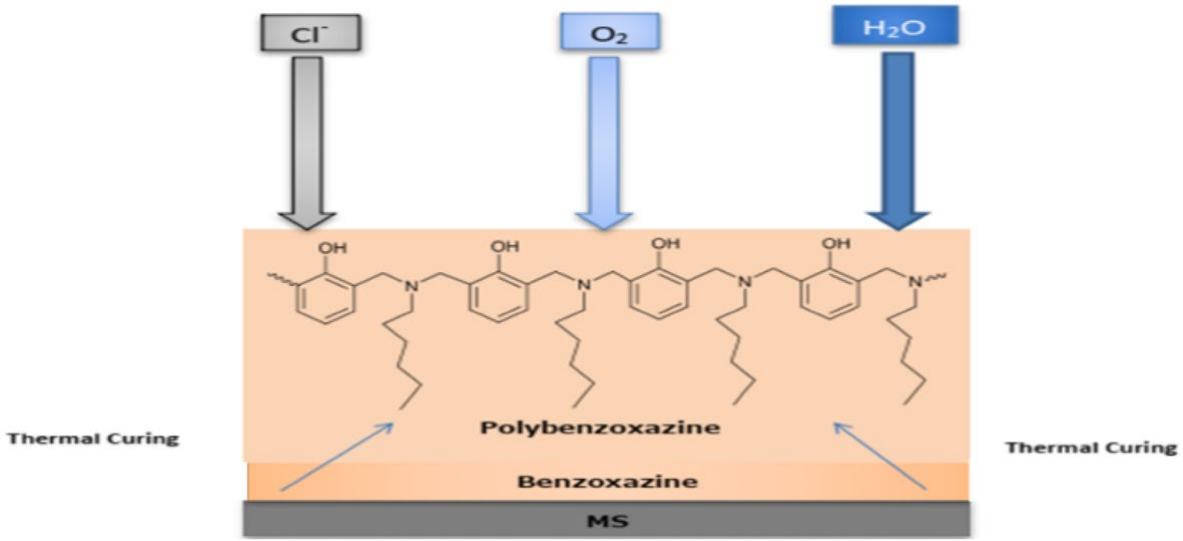
Corrosion protection mechanism of MS coated with poly(SA-Hex-BZ) as anticorrosion coating.

The SA-Hex-BZ precursor was capable of forming a compact crosslinking network after thermal curing. As such coating was completely cured at 210 °C for 2 h and exhibited hydrophobic qualities to produce poly(SA-Hex-BZ), which considerably improved the corrosion resistance of MS. Figure 14 indicates that the barrier ability was principally responsible for the poly(SA-Hex-BZ) coating's anti-corrosive property after curing. The hydrophobic poly(SA-Hex-BZ) coating with a dense crosslinked network can enhance corrosion reaction barrier ability and reduce corrosive medium penetration compared to hydrophilic bare MS. From Table 5, it can be noticed that our polymer named poly(SA-Hex-BZ) has the highest inhibition effect from other different inhibitors in the same corrosive medium.

**Table 5:** Shows the comparison between the inhibition efficiency (IE%) of some reported inhibitors in the same corrosive medium (H_2_SO_4_).

	Inhibition efficiency (IE%)	References
Poly(SA-Hex-BZ)	91.7	This study
1-Methyl-3-propylimidazolium iodide (MPII)	91	^39^
Solanum tuberosum (ST)	90.8	^40^
Novel polythiadiazole, namely poly[(2,6-dicarbonylpyridine)(2,5-dihydrazinyl-1,3,4-thiadiazole)] (AMTP)	90.3	^41^
Poly(o-methoxy-aniline) (PMA)	85.0	^42^
Polyamino-benzoquinone (PAQ)	85.6	^43^
polyethylene glycol methyl ether (PEGME)	84.2	^44^
Tetradenia riparia (Tr)	80.4	^45^
2-Mercaptobenzothiazole (MBT)	78.0	^46^
Cefuroxime	77.6	^47^
Hydroxyethyl cellulose (HEC)	73.1	^48^
Polyacrylamide (PA)	67.8	^49^
Polyvinylpyrolidone (PVP)	58.0	
A benzenesulfonamide-based benzoxazine compound (BSB)	59.8	^50^
Polyethylene glycol (PEG)	40.2	^51^
Polyvinyl alcohol (PVA)	36.3	

## Conclusions

In summary, the SA-Hex-BZ monomer was prepared through three steps, including condensation, reduction, and ring-closing reaction, as displayed in Fig. 1. DSC and TGA analyses revealed that the poly(SA-Hex-BZ) produced following the thermal curing of SA-Hex-BZ at 210 °C had a high value of T_*g*_ and char yield due to the higher crosslinking density and degree of intramolecular hydrogen bonding in poly(SA-Hex-BZ). In 0.1 M H_2_SO_4_ solution, MS coated with poly(SA-Hex-BZ) revealed superior resistance to corrosion than bare MS. This emerged from coordination interaction among poly(SA-Hex-BZ) coatings and MS surfaces, that have high adsorbing properties. It can be noticed that our polymer named poly(SA-Hex-BZ) has the highest inhibition effect. Finally, incorporating the hexyl group into benzoxazine coatings appears to be a great option to provide high-performance corrosion protection.

## Supplementary Information

Supplementary Information.

## Data Availability

All data generated or analyzed during this study are included in this published article.
