# Supplementary material for: Synthesis, characterization and protective efficiency of novel polybenzoxazine precursor as an anticorrosive coating for mild steel
Source: Sci Rep. 2023 Apr 5;13:5581. doi: 10.1038/s41598-023-30364-x (PMC10076265; doi:10.1038/s41598-023-30364-x)
Supplement: Supplementary file 1 — Supplementary Information. [file 41598_2023_30364_MOESM1_ESM.doc]

**Supporting Information for**

**Synthesis, Characterization and Protective Efficiency of Novel Polybenzoxazine Precursor as an Anticorrosive Coating for Mild Steel**

**Ahmed M. M. Soliman^1^, Kamal I. Aly^2*^, Mohamed Gamal Mohamed^2,3,*^, Amer A. Amer^1^, Mostafa R. Belal^1^, and Mohamed Abdel-Hakim^4^**

^1^ Department of Chemistry, Faculty of Science, Sohag University, Sohag 82524, Egypt.

^2^ Polymer Research Laboratory, Chemistry Department, Faculty of Science, Assiut University, Assiut 71516, Egypt.

^3^ Department of Materials and Optoelectronic Science, Center for Functional Polymers and Supramolecular Materials, National Sun Yat-Sen University, Kaohsiung, Taiwan.

^4^ Chemistry Department, Faculty of Science, Al-Azhar University, Assiut 71524, Egypt.

Corresponding authors:

E-mail: [kamalaly@aun.edu.eg](mailto:kamalaly@aun.edu.eg)(K.I.Aly)&[mgamal.eldin12@aun.edu.eg](mailto:mgamal.eldin12@aun.edu.eg)(M.G.Mohamed)

**Figure S1**. Raman spectrum of poly(SA-Hex-BZ).


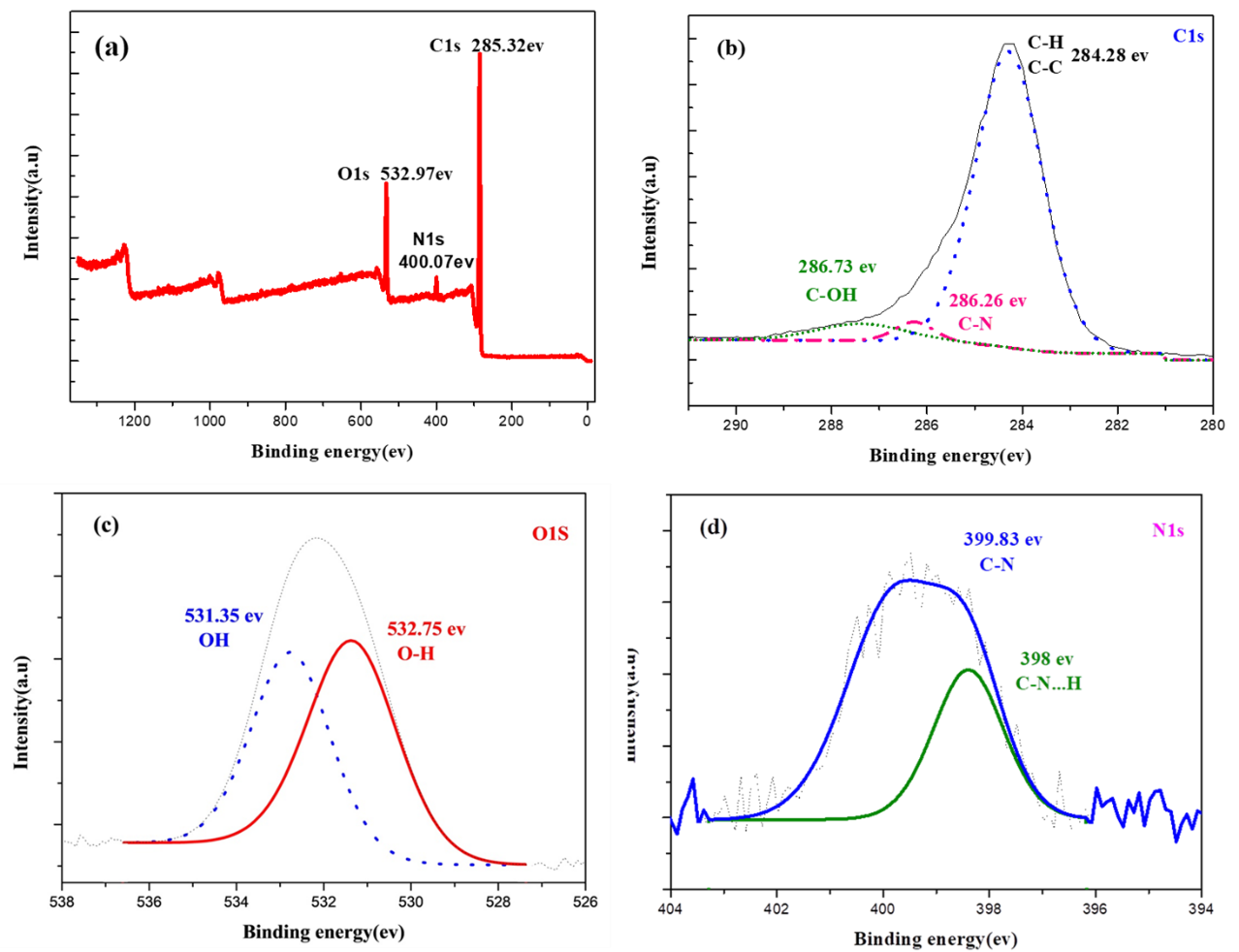


**Figure S2**. XPS spectrum of poly(SA-Hex-BZ).

**Figure S3**. Nyquist plots of uncoated MS and MS coated with poly(SA-Hex-BZ) after thermal curing at 210 ^o^C for 2 h.


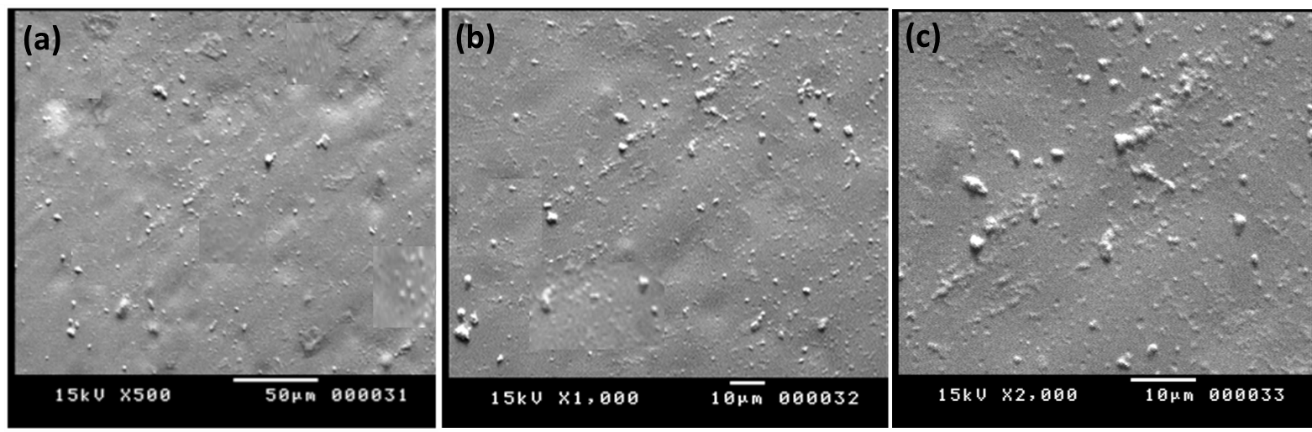


**Figure S4**. SEM surface images of coated MS with poly(SA-Hex-BZ) at different magnifications (X500 (a), X1,000 (b) and X2,000 (c)) after the corrosion process.

.
